# Case Report: Nasal swelling and ulceration as a unique immune-related cutaneous adverse event of atezolizumab in small cell lung cancer

**DOI:** 10.3389/fimmu.2026.1818537

**Published:** 2026-04-30

**Authors:** Junhui Yang, Xuemei Du, Hongyun Liu, Yueqin Hao

**Affiliations:** 1 School of Clinical Medicine, Shandong Second Medical University, Weifang, China; 2 Department of Thoracic Medical Oncology, Qingdao Municipal Hospital, Qingdao, China; 3 Department of Pathology, Qingdao Municipal Hospital, Qingdao, China

**Keywords:** atezolizumab, immune-related adverse events (irAEs), immune-related cutaneous adverse events (ircAEs), rare case, small cell lung cancer (SCLC)

## Abstract

This article reports a rare immune-related cutaneous adverse event (ircAE) manifesting as nasal swelling and ulceration in a patient with small cell lung cancer (SCLC) after treatment with atezolizumab. A patient with extensive-stage SCLC developed symptoms of nasal swelling and ulceration during treatment with atezolizumab combined with anlotinib. Based on clinical test results, we ruled out infections and autoimmune diseases. Histopathological analysis of the lesion biopsy excluded metastatic carcinoma. According to the patient’s medication history, we considered that the nasal swelling and ulceration were extremely rare ircAEs caused by atezolizumab. After we discontinued immunotherapy and administered glucocorticoids, the patient’s nasal skin symptoms improved significantly. We searched multiple medical databases such as PubMed, but did not find similar reports.

## 1 Introduction

Atezolizumab is a programmed death-ligand 1 (PD-L1) immune checkpoint inhibitor that reverses functional exhaustion of T cells in the tumor microenvironment and restores their ability to recognize, infiltrate, and kill tumor cells (1). Widely used in the treatment of various malignancies, atezolizumab inevitably impairs the body’s autoimmune tolerance to peripheral tissues while breaking tumor immune tolerance, thereby triggering a series of immune-related adverse events (irAEs) (2). Among irAEs induced by immune checkpoint inhibitors, ircAEs are relatively common (3, 4), but ircAEs manifested as nasal ulceration and swelling are extremely rare. This paper presents a case of a 56-year-old male patient with small-cell lung cancer who developed a rare ircAE involving the nasal skin following treatment with atezolizumab. The purpose of this case report is to draw clinicians’ attention to rare ircAEs and to avoid clinical misdiagnosis and mistreatment.

## 2 Case description

**Figure 1:**
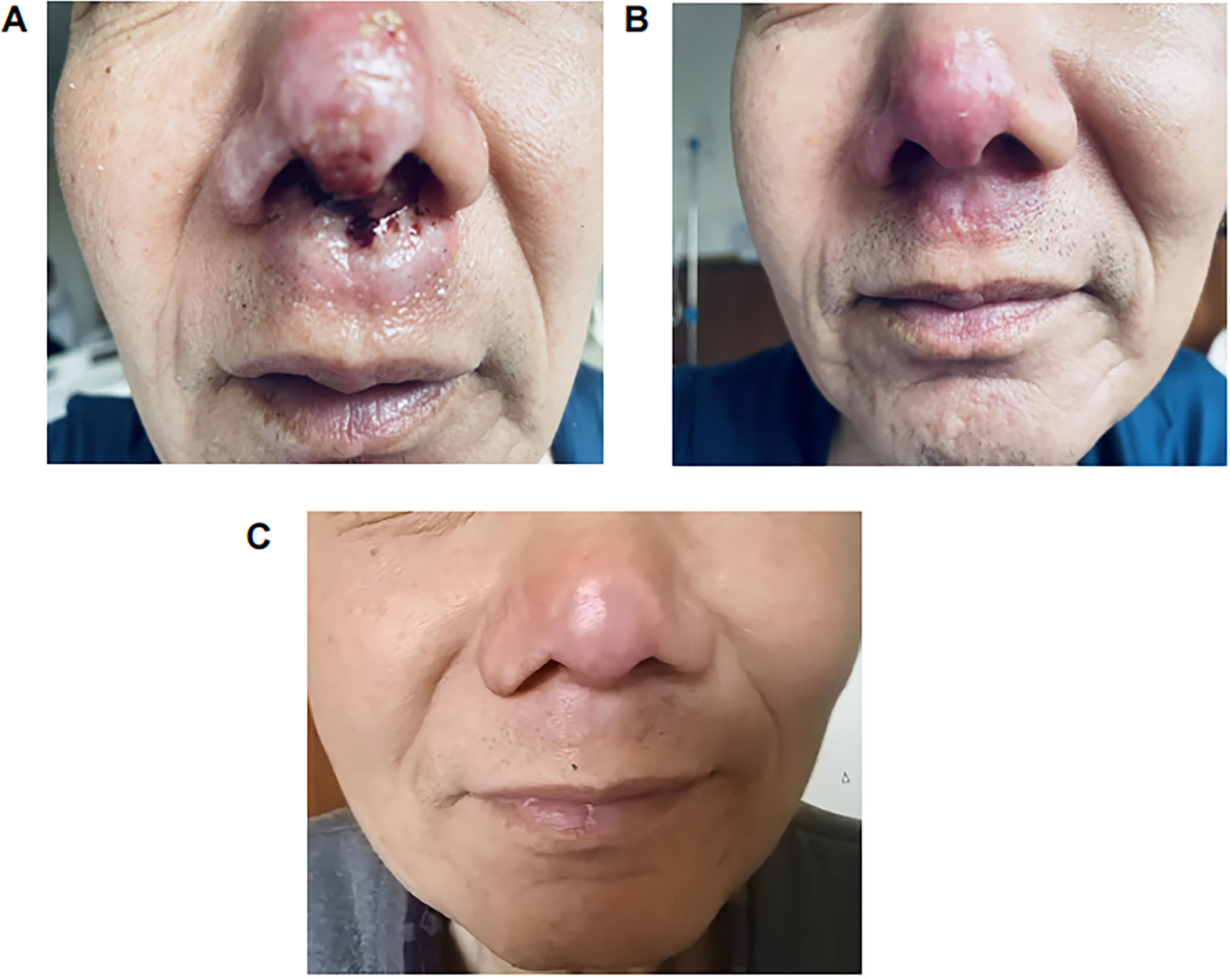
**(A)** Baseline (onset of nasal skin lesions), the skin on the dorsal side of the nose, the right alar of the nose, the tip of the nose and the base of the nose is swollen, ulcerated, and scabbed. **(B)** Two weeks after discontinuation of atezolizumab and initiation of glucocorticoid therapy, the patient’s nasal skin improved significantly. **(C)** 6 months post-discontinuation of atezolizumab, the patient’s nasal skin had basically returned to normal.

A 56-year-old male with a two-year history of extensive-stage SCLC complained of nasal ulceration for more than 2 months and was admitted to Qingdao Municipal Hospital on June 7, 2025. Two months ago (April 2025), the patient developed swelling, bleeding, and ulceration of the skin at the tip of the nose without an obvious cause, accompanied by pain and itching. Topical medication did not improve the condition, and the swelling and ulceration of the local skin worsened. The patient denied a history of smoking, alcohol consumption or other unhealthy habits. He had no history of drug allergy, surgery, familial malignancy or hereditary diseases, and no other previous medical illnesses. The patient is an office clerk and maintains a harmonious family relationship. The skin on the dorsal side of the nose, the right nasal ala, the tip of the nose and the base of the nose was swollen, ulcerated, and scabbed (**Figure 1A**). Physical examination showed no abnormalities in the heart or lungs. The patient’s blood routine, liver function, kidney function, and coagulation function tests were all normal. Anti-neutrophil cytoplasmic antibodies (ANCA) and anti-nuclear antibody (ANA) profiles were negative.

**Figure 2:**
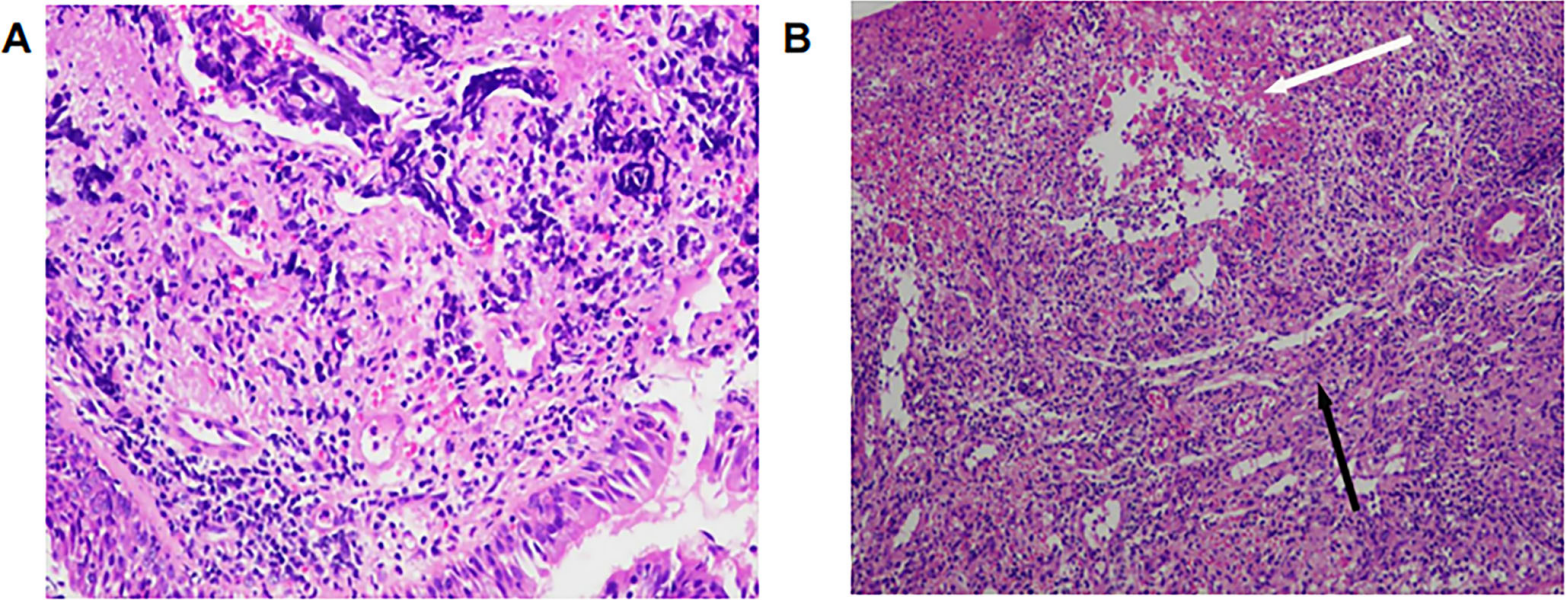
**(A)** The pathological sections showed severe chronic inflammation (HE ×200). **(B)** The pathological sections showed tissue degeneration, necrosis, and granulation tissue formation. The white arrow indicates tissue degeneration and necrosis, while the black arrow indicates inflammatory granulation tissue (HE ×100).

**Figure 3:**
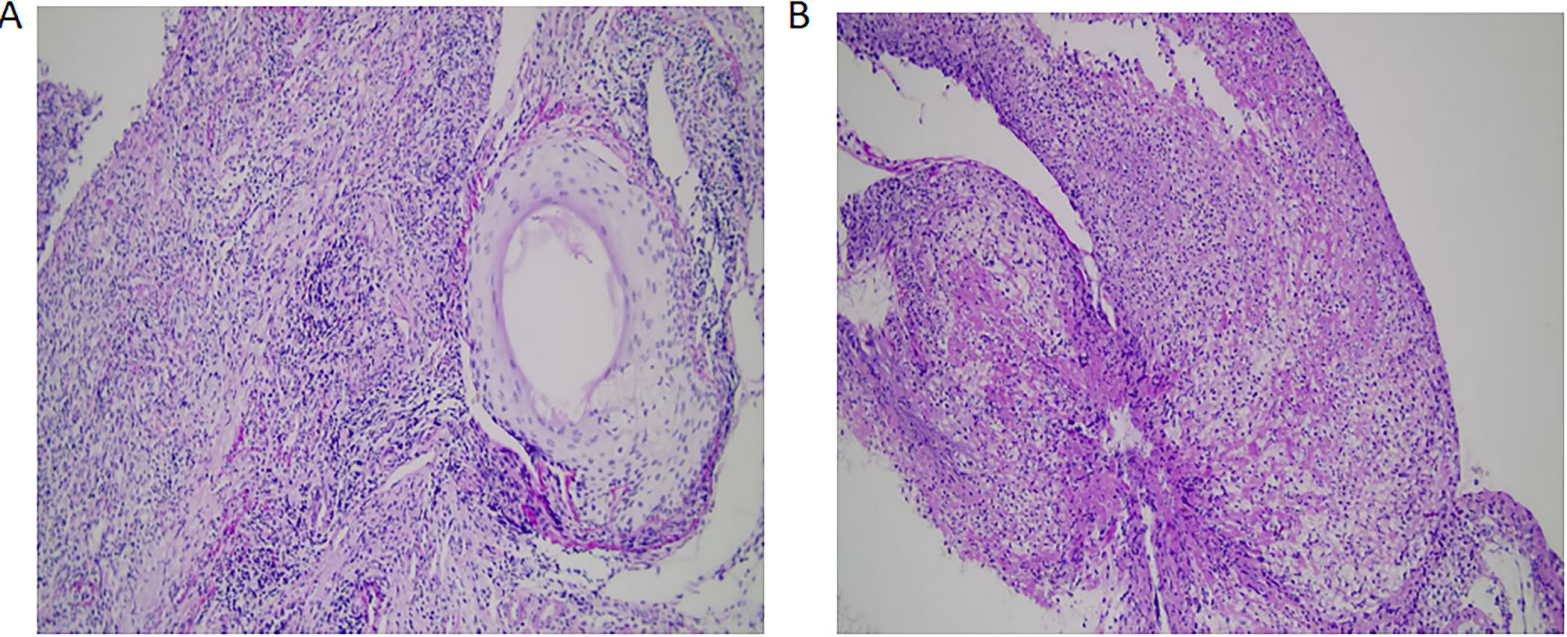
**(A)** PAS staining: negative ×200. **(B)** Acid-fast staining: negative ×200.

**Figure 4:**
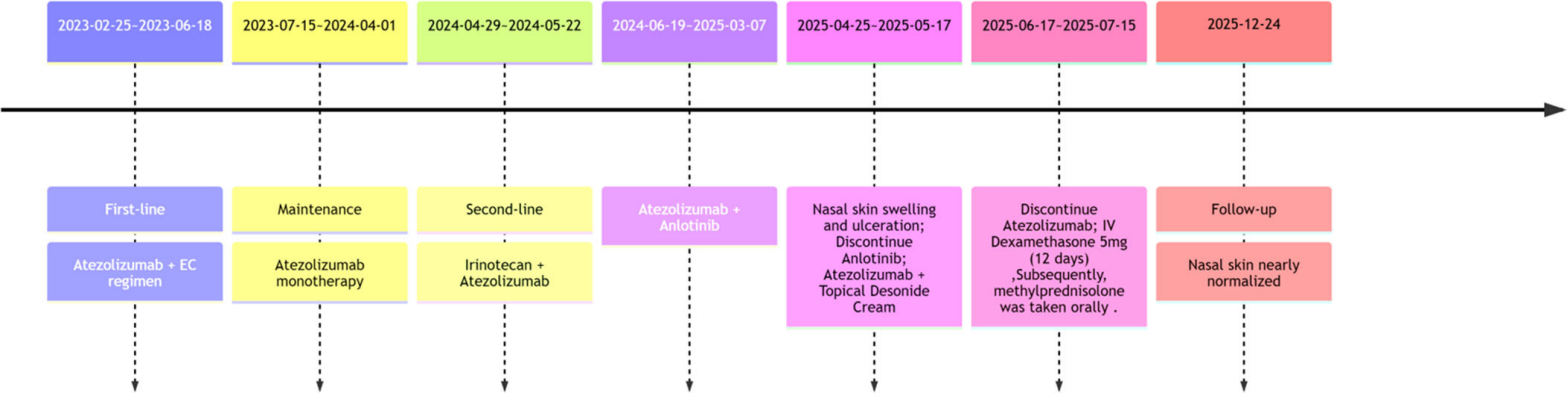
Timeline of atezolizumab administration and the occurrence, management, and outcome of cutaneous immune-related adverse events. The patient was diagnosed with extensive-stage small cell lung cancer (ES-SCLC) and subsequently received long-term treatment with atezolizumab. In April 2025, the patient presented with nasal skin swelling and ulceration. Atezolizumab was discontinued in June 2025, followed by intravenous administration of dexamethasone sodium phosphate and oral methylprednisolone tablets. The patient’s nasal skin lesions gradually improved. At follow-up through December 2025, the patient’s nasal skin condition was nearly normal.

The patient was diagnosed with extensive-stage SCLC on February 18, 2023, accompanied by metastases to the right supraclavicular lymph nodes, pleura, and brain. On February 25, 2023, according to the NCCN guidelines, atezolizumab combined with the EC regimen (etoposide and carboplatin) was administered as the first-line treatment for 6 cycles. Subsequently, atezolizumab was used for maintenance treatment. On April 19, 2024, chest CT showed disease progression. The second-line treatment was given with irinotecan combined with atezolizumab for two cycles. In June 2024, due to the progression of brain metastases, oral anlotinib combined with atezolizumab was administered as the third-line treatment. In April 2025, the patient presented with swelling and ulceration of the nasal skin. Initially, it was considered that the nasal swelling and ulceration might be related to anlotinib, so anlotinib was discontinued and atezolizumab monotherapy was continued. By June 2025, anlotinib had been discontinued for two months, and the patient’s nasal lesions continued to worsen. During this period, empiric broad-spectrum antimicrobial therapy also showed no efficacy. Therefore, anlotinib-related adverse reactions and infectious etiologies were essentially ruled out. Under these circumstances, we conducted multidisciplinary consultations with dermatologists and otorhinolaryngologists. Following comprehensive physical examination, medical history inquiry and review of the treatment course, the consulting physicians recommended local biopsy to exclude lung cancer metastasis and herpesvirus infection. Biopsy of the nasal lesion was performed on June 11, 2025. Histopathological examination showed diffuse mixed inflammatory cell infiltration with a predominance of lymphocytes, along with tissue degeneration, necrosis and granulation tissue formation. No findings consistent with vasculitis were detected. The immunohistochemical results showed CK (focal+, representing residual normal epidermal basal cells), p63 (-), and CD56 (-) (**Figures 2A, B**). The infiltrating lymphocytes are predominantly T lymphocytes (diffusely positive for CD3), exhibiting a diffuse sheet-like distribution. They consist of a mixed population of CD4-positive and CD8-positive T cells, with CD8 expression being slightly more abundant than CD4. The acid-fast staining and Periodic Acid-Schiff (PAS) staining of the pathological sections were both negative (**Figures 3A, B**). Taking into account the patient’s medication history and pathological biopsy results, we considered that the most likely cause of nasal swelling was the ircAEs caused by atezolizumab. Therefore, atezolizumab treatment was discontinued, and at the same time dexamethasone 5 mg was intravenously injected for 12 days. After adjusting the medication, the patient’s nasal skin improved significantly (**Figure 1B**). Subsequently, low-dose glucocorticoids were administered orally for one month. The patient was followed up monthly at the outpatient clinic, and repeated examinations of inflammatory markers, liver function and renal function showed no significant abnormalities. He was followed up until December 24, 2025, and his nasal skin had almost completely returned to normal (**Figure 1C**). The authors constructed a timeline diagram to present the patient’s prior oncological treatment regimens, as well as the management and follow-up of cutaneous adverse events, thereby providing a clearer overview of the clinical course (**Figure 4**).

## 3 Patient perspective

As a 56-year-old male with a history of lung cancer for more than two years, I have been receiving consistent treatment at the Qingdao Municipal Hospital. In April 2025, I suddenly developed nasal skin ulceration, which showed no improvement with topical medication administration and filled me with intense worry and anxiety. I was particularly concerned that this symptom might be indicative of tumor metastasis. Following targeted clinical treatment, my nasal symptoms have completely resolved and the skin has returned to near normal. I would like to express my sincere gratitude to the hospital and all the medical staff for their professional diagnosis, effective treatment and thoughtful care.

## 4 Discussion

This case demonstrates an extremely rare manifestation of an immune-related adverse reaction to atezolizumab, which manifests as swelling, ulceration and bleeding of the nasal skin. Its clinical manifestations are completely different from common ircAEs such as maculopapular rashes and pruritus (5), and are highly misleading. Clinically, it is easily misdiagnosed as metastatic cancer. Atezolizumab, as an immune checkpoint inhibitor (ICI) of PD-L1, can cause irAEs that involve multiple organ systems throughout the body (2, 3, 5). Common clinical manifestations of ircAEs include maculopapular rashes, pruritus, psoriasis-like lesions, eczema and lichenoid skin diseases (6), while rare clinical manifestations include bullous pemphigoid, vitiligo-like skin lesions, alopecia and other lesions (3, 4). We searched multiple databases including PubMed, Wanfang Data, China National Knowledge Infrastructure (CNKI), and VIP Chinese Science and Technology Journal Database with the keywords atezolizumab-associated nasal ulcer and PD-L1 inhibitor-related nasal ulcer, and no relevant reports were found. This patient was diagnosed with extensive-stage SCLC. According to the NCCN guidelines (7), chemotherapy combined with atezolizumab was administered as the first-line treatment. The patient experienced extremely rare irAEs manifesting as nasal swelling and ulceration during third-line treatment with anlotinib combined with atezolizumab. In clinical practice, the rare ircAEs should mainly be differentiated from the following diseases. First, local skin infections, including bacterial, herpesviral or fungal infections (8). Second, primary or secondary tumors may also present as swelling and ulceration of the local skin (9). Third, autoimmune diseases, such as granulomatosis with polyangiitis (GPA, formerly Wegener’s granulomatosis) and systemic lupus erythematosus (SLE), can also induce cutaneous lesions (10, 11). In addition, adverse reactions of other drugs should also be excluded (12). Therefore, clinicians need to carefully inquire about the patient’s medical history, symptoms, and medication history. When differentiation is difficult, skin biopsy should be considered to confirm the diagnosis. We initially suspected that anlotinib was responsible for the patient’s nasal mucosal and cutaneous lesions. However, the symptoms showed no obvious improvement two months after anlotinib was discontinued. The patient’s hepatic and renal functions were normal in this case. Given the biological half-life of anlotinib, the cutaneous ulceration is highly inconsistent with anlotinib-induced toxicity (13). During this period, empirical antibiotic treatment showed no efficacy. Acid-fast staining and PAS staining were both negative. These findings provide no evidence for bacterial, fungal, or mycobacterial infections. The patient presented with nasal cutaneous ulceration persisting for several months, in the absence of clustered vesicles. Histopathological examination demonstrated predominant lymphocytic infiltration, with no viral inclusions or ballooning degeneration identified (14). The lesion showed a favorable response to glucocorticoid therapy, thus excluding herpesvirus infection. The biopsy of nasal tissue showed inflammatory changes, ruling out the possibility of metastatic cancer. The blood ANA profile and ANCA were both negative, which did not support an autoimmune etiology. Therefore, we considered that the most likely cause of nasal swelling was irAEs induced by atezolizumab.

This case report presents several prominent clinical strengths. First, it documents a rare case of atezolizumab-induced ircAEs manifesting as nasal skin lesions in a patient with SCLC, which fills the gap in clinical reports on rare ircAEs associated with immune checkpoint inhibitors. Second, the diagnostic and therapeutic process of this case is complete with a comprehensive differential diagnosis: based on the patient’s auxiliary examination results and treatment course, infection, tumor metastasis and autoimmune diseases were systematically ruled out, which ensured the accuracy of the diagnosis of atezolizumab-related ircAEs. Third, a detailed timeline was constructed to clearly illustrate the administration course of atezolizumab, the onset of cutaneous adverse events as well as the subsequent diagnosis and treatment of the condition, and a sufficiently long-term follow-up was conducted until December 2025 to confirm the therapeutic outcome. Nevertheless, this study has several inherent limitations. First, although we attributed the nasal mucosal ulceration to an immune-related adverse event induced by atezolizumab based on the clinical timeline and the favorable response to glucocorticoid therapy, the possibility of a delayed effect of anlotinib, or synergistic toxicity between anlotinib and atezolizumab, cannot be completely ruled out. Second, FoxP3 detection was not available at our institution, and thus no results for this marker were obtained.

## 5 Conclusion

The diagnosis and treatment process of this case suggests that clinicians need to closely monitor the patient’s skin changes during atezolizumab treatment. For severe skin-related adverse events, especially when they occur in rare areas, consultations with multidisciplinary experts such as dermatologists, oncologists, and rheumatologists should be conducted. Once moderate to severe ircAEs are diagnosed, glucocorticoids are usually the preferred systemic treatment. The initial dose and course of treatment should be adjusted according to the severity of ircAEs (15). In addition, immune-related adverse events often involve multiple organs and systems. Clinical assessment should not be limited to skin involvement but should also include the evaluation of potential complications in the heart, lungs, endocrine system, and digestive system. Therefore, establishing a comprehensive adverse event monitoring and reporting system is conducive to the timely identification and management of novel or rare irAEs. Future multicenter, large-sample clinical studies and mechanistic research are warranted to further investigate such rare ircAEs, thereby providing evidence-based clinical guidance for the rational clinical application of immune checkpoint inhibitors and the standardized management of ircAEs arising during treatment.

## Data Availability

The original contributions presented in the study are included in the article/supplementary material. Further inquiries can be directed to the corresponding author.
